# Assay for ADAMTS-13 Activity with Flow Cytometric Readout

**DOI:** 10.1021/acsomega.2c02077

**Published:** 2022-08-24

**Authors:** Jens Müller, Nasim Shahidi Hamedani, Hannah L. McRae, Heiko Rühl, Johannes Oldenburg, Bernd Pötzsch

**Affiliations:** Institute of Experimental Hematology and Transfusion Medicine, University Hospital Bonn, Bonn 53127, Germany

## Abstract

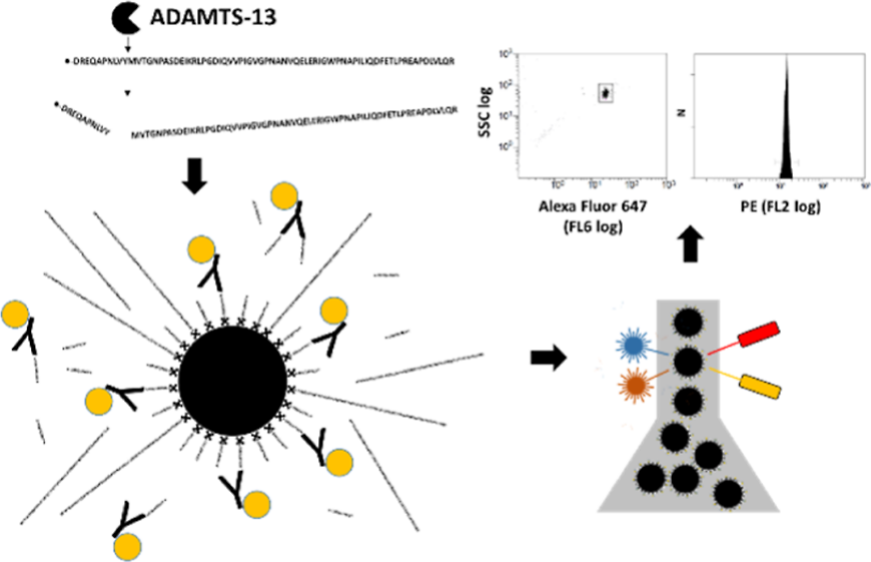

A disintegrin and metalloproteinase with a thrombospondin type 1 motif, member 13 (ADAMTS-13) is a metalloprotease that regulates the size of circulating von Willebrand factor (vWF) multimers. Severe lack of ADAMTS-13 activity [<10% of normal (0.1 IU/mL)] leads to thrombotic thrombocytopenic purpura (TTP), a specific type of thrombotic microangiopathy (TMA). Timely determination of plasma ADAMTS-13 activity is essential to discriminate TTP from other types of TMA with respect to adequate treatment. Identification of the minimal substrate motif for ADAMTS-13 within the A2 domain of vWF (vWF73) as well as the generation of monoclonal antibodies (mAbs) that specifically recognize the ADAMTS-13 cleavage site enabled the development of a variety of methods for determination of plasma ADAMTS-13 activity. In order to further extend the range of analytical platforms applicable for quantitative determination of plasma ADAMTS-13 activity, a specific, vWF/mAb-based assay with flow cytometric readout was developed and validated. Basic assay characteristics include a total assay time of 80 to 90 min, a near linear dynamic range from 0.005 (lower limit of quantification) to 0.2 IU/mL, and intra- and interassay coefficients of variation below 5 and 30% at input plasma ADAMTS-13 activities of 0.015 and ≤0.050 IU/mL, respectively. When compared to the results obtained with a commercially available quantitative ADAMTS-13 activity ELISA, analysis of 18 plasma samples obtained from patients with suspected TTP revealed full agreement of results with respect to the clinical 0.1 IU/mL TTP threshold. Based on these data, it is assumed that the described assay principle can be successfully transferred to virtually all laboratories that have a flow cytometer available.

## Introduction

ADAMTS-13 (a disintegrin and metalloproteinase with a thrombospondin type 1 motif, member 13), also known as von Willebrand factor (vWF)-cleaving protease, is a plasma glycoprotein that regulates the size distribution of vWF, a protein that is synthesized as ultralarge multimers (ULVWF) in megakaryocytes and endothelial cells.^1^ When exposed to high shear stress, for example, during endothelial secretion, vWF unfolds, allowing cleavage by ADAMTS-13 within the A2 domains at random positions of the ULVWF.^2−5^ Regulation of the vWF size is critical for its function since ULVWF induces platelet aggregation in the absence of local shear stress.^3,6,7^ Accordingly, severe lack of ADAMTS-13 activity, either due to hereditary forms or, as in >95% of cases, acquired (e.g., due to the formation of autoantibodies), results in spontaneous assembly of ULVWF–platelet complexes. This leads to a thrombotic complication termed thrombocytopenic purpura (TTP), a specific type of thrombotic microangiopathy (TMA).^8,9^

Differentiation of TTP from other subtypes of TMA, especially the (atypical) hemolytic uremic syndrome [(a)HUS], is crucial for early treatment decisions.^10^ In contrast to (a)HUS, acquired TTP necessitates therapeutic plasma exchange (TPE) in order to replace ADAMTS-13 and remove potentially present anti-ADAMTS-13 autoantibodies.^11^ In addition, caplacizumab, a bivalent variable-domain-only anti-vWF immunoglobulin fragment that inhibits the interaction between the ULVWF A1 domains and platelets, has become an additional treatment option.^12^ In hereditary TTP, also known as Upshaw–Schulman syndrome, recombinant ADAMTS-13 is set to become the treatment of choice.^13^ Clinical and laboratory features of TTP include fever, renal dysfunction, hemolysis, blood smear schistocytes, and thrombocytopenia.^8,14^ While these findings may justify the start of TPE for suspected TTP, only a low plasma ADAMTS-13 activity level (<10% of normal [<0.1 IU/mL]) confirms the diagnosis and indicates continuation of treatment.^15^ Accordingly, timely determination of plasma ADAMTS-13 activity is essential in TMA classification.^16^

Classically, vWF has served as the substrate in ADAMTS-13 functional assays. In the absence of shear stress, vWF is unfolded by a denaturing agent (typically urea) under low-salt concentrations and ADAMTS-13 activated by addition of divalent cations, usually Ba^2+^.^17,18^ After incubation, ADAMTS-13-mediated cleavage of vWF is assessed by electrophoretic analysis or via functional or immunological vWF assays.^18−20^ In an attempt to improve this kind of assay, Cruz et al. applied a recombinant 25 kDa vWF A2 domain polypeptide construct as the substrate, allowing for cleavage by ADAMTS-13 under nondenaturing conditions.^21,22^ Afterward, Kokame et al. described the minimal substrate for ADAMTS-13 (vWF73) that comprises vWF A2 amino acids D1596 to R1668.^23^ This group also developed a fluorescence resonance energy transfer (FRET) substrate for determination of ADAMTS-13 activity based on this peptide (FRETS-vWF73).^24^ Eventually, Kato et al. generated a panel of monoclonal antibodies (mAbs) that specifically recognize Y1605, which is the C-terminal vWF A2 residue after ADAMTS-13 cleavage. Based on vWF73, the group used one of these mAbs for the development of an enzyme-linked immunosorbent assay (ELISA) for plasma ADAMTS-13 activity.^25^

The minimal substrate motif vWF73 along with ADAMTS-13 cleavage-specific mAbs represent a versatile toolbox for plasma ADAMTS-13 analysis. At present, most commercially available quantitative assays for determination of plasma ADAMTS-13 activity are based on these molecules, either in homogeneous FRET- or in heterogeneous mAb-based configuration.^26−30^ However, these assays all require specific equipment such as microtiter plate washers, microtiter plate compatible fluorescence readers, and/or random access analyzers. With respect to the low incidence of the disease, these constraints make it logistically and financially difficult to establish the described assays in routine clinical laboratories.^31^ As a result, patient samples are typically shipped to specialized laboratories for ADAMTS-13 testing. This requires sufficient logistic infrastructure, has significant preanalytic implications, and subsequently results in (often critical) delays in the availability of test results.^31,32^

To address this problem, a rapid, semiquantitative plasma ADAMTS-13 assay based on flow-through technology was previously described and introduced in the market.^33^ A multicenter study compared this assay to a quantitative ADAMTS-13 activity ELISA. Considering a plasma ADAMTS-13 activity level of 0.1 IU/mL as the clinical threshold, the study revealed positive and negative predictive values (NPVs) of 75 and 96%, respectively. Overall, 10% of samples analyzed in the study were misclassified by the rapid assay with respect to the clinical threshold.^34^ Although especially the high NPV may warrant the application of this assay, at least positive results (plasma ADAMTS-13 activity found to be within the 0 or 0.1 IU/mL assay categories) still necessitate subsequent quantitative analysis for confirmation.

Instead of acquisition of novel analyzers or laboratory equipment, pre-established analytical platforms in conjunction with appropriate assay design may be applied for the analysis of plasma ADAMTS-13 activity. In line with this strategy, the adaptation of a vWF73/mAb-based assay with a flow cytometric readout is described in the present paper.

## Results and Discussion

### General Assay Principle and Evaluation of Assay Performance

**Figure 1:**
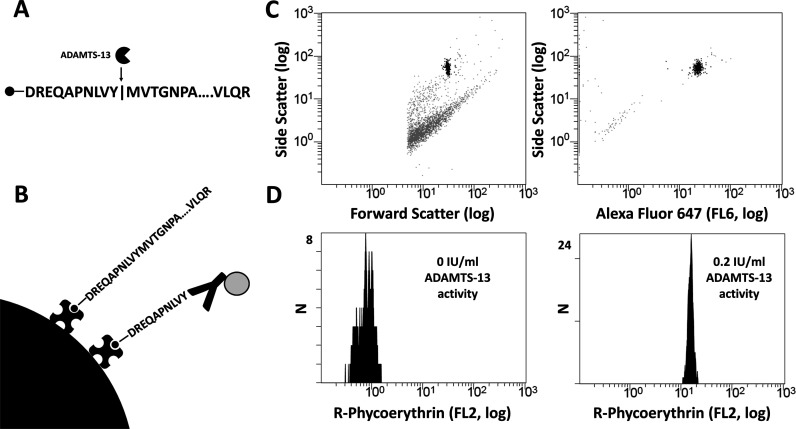
Assay principle. (A) The plasma sample is diluted 1 in 10 in substrate solution (5 mM BaCl_2_) containing 316 nM biotinylated (●-) vWF-substrate (vWF73) and incubated for 30 min at 37 °C under shaking. (B) After stopping of the reaction with EDTA, samples are diluted and Alexa Fluor 647-labeled, and streptavidin-coated beads as well as a PE-labeled mAb (Y○) that binds to the ADAMTS-13 cleavage site (PE–mAb) are added. (C) After incubation, samples are further diluted and Alexa Fluor-labeled beads gated by FSC/SSC (left panel) or fluorescence (Alexa Fluor 647, FL6, right panel) emission characteristics. (D) Corresponding MFI values (R-PE, FL2) represent the ADAMTS-13 activity in the original plasma sample (data of plasma calibrators shown).

The general principle of the proposed assay is schematically presented in Figure 1. Briefly, this is a flow cytometric assay comprised of an enzyme (plasma ADAMTS-13)/substrate (biotinylated vWF73) reaction, followed by R-phycoerythrin (PE)–mAb-based detection of ADAMTS-13 mediated cleavage of substrate after binding to streptavidin-coated beads (beads).

**Figure 2:**
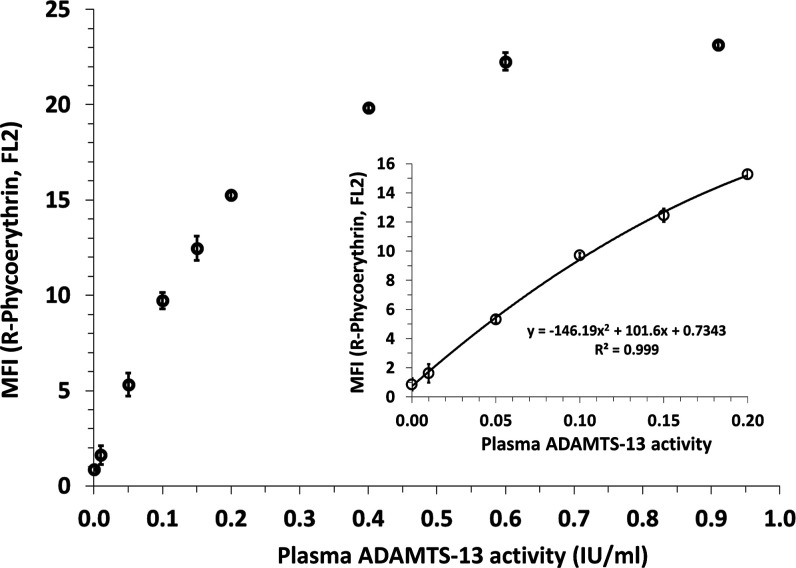
General assay performance. A total of nine different plasma ADAMTS-13 calibrators covering a range from 0 to 0.91 IU/mL were prepared by diluting the WHO international standard for plasma ADAMTS-13 activity in heat-inactivated PNP. All calibrators were introduced to three independent experiments, and the corresponding results are shown as MFIs ± SD that reflect binding of the R-PE-labeled mAb used for assay readout. The small figure shows the clinically relevant lower concentration range (0 to 0.2 IU/mL).

Analysis of prepared ADAMTS-13 plasma calibrators (*n* = 9; 0 to 0.91 IU/mL) in three independent runs [1× using unlabeled beads, 2× using Alexa Fluor 647 (AF647)-labeled beads for assay readout] demonstrated that AF647-labeled beads could be easily identified by forward-/side-scatter (FSC/SSC) or, more distinct from background, AF647 (FL6) fluorescence emission characteristics (compare Figure 1C). The measured mean [± standard deviation (SD)] fluorescence intensities (MFIs) that reflect binding of the PE-labeled mAb (FL2, compare Figure 1D) are shown in Figure 2.

The data demonstrate that the assay has a high reproducibility and sensitivity, while both unlabeled and AF647-labeled beads became equally saturated with respect to ADAMTS-13-cleaved, biotinylated vWF73/PE–mAb complexes. This resulted in lower correlation between plasma ADAMTS-13 activity and observed MFI (PE) values from levels higher than 0.2 IU/mL. The number of biotinylated vWF73 molecules (∼1.9 × 10^11^) during loading of the AF647-labeled beads (∼10,000) was chosen to be ∼10-fold higher than the theoretical binding capacity stated by the manufacturer [Dynabeads M-280 streptavidin: ∼200 pmol of biotinylated peptides per mg (∼6.5 × 10^7^ beads), corresponding to ∼1.8 × 10^6^ peptides/bead]. Accordingly, given the high affinity between biotin and streptavidin, one must assume that AF647-labeled beads were fully loaded with (ADAMTS-13-cleaved) biotinylated vWF73 during flow cytometric analysis. Thus, it appears likely that the observed flattening of the MFI (PE) signal is primarily caused by high substrate cleavage rates in combination with limited association of PE–mAb with ADAMTS-13-cleaved biotinylated vWF73 on AF647-labeled beads. Furthermore, the amount of beads used, a relatively low degree of PE-labeling (Figure S1), and the size of the PE–mAb (0.66 pmol/reaction, >250 kDa, Figure S1) in combination with the small surface of the AF647-labeled beads (2.8 μm diameter) may have played a role. In general, no agglutination of AF647-labeled beads was observed even at high levels of plasma ADAMTS-13 activity (Figure S2). Interestingly, experimental application of streptavidin-coated, fluorescent microparticles with higher diameters (3.5 and 5.7 μm) that were explicitly intended for flow cytometric analysis clearly showed inferior results under comparable assay conditions (Figure S3). It therefore appears that the obviously higher streptavidin density on the AF647-labeled beads outweighed their smaller diameter with respect to overall assay performance. Therefore, AF647-labeled beads were used for all further experiments.

One of the key advantages of plasma ADAMTS-13 activity analysis by flow cytometry is that the described assay has a potentially high specificity while exhibiting the beneficial characteristics of a homogenous assay format. Indeed, signal generation is not directly due to FRET-substrate cleavage in the patient plasma matrix but rather based on more specific detection of the ADAMTS-13 cleavage site by the mAb used.^35^ Indeed, parallel analysis of patient plasma samples with low ADAMTS-13 activities (<0.2 IU/mL) in the absence or presence of a protease inhibitor mix (EDTA-free, therefore not inhibiting metalloproteinases) yielded comparable quantitative results (Figure S4). The assay is also not influenced by fluorescence-quenching agents such as bilirubin, which is potentially present in patient plasma samples, thereby eliminating a potential preanalytical error.^36^ Furthermore, in contrast to ELISA-based assays, no washing step is needed during application of the flow cytometric assay.

Due to the observed high assay sensitivity and near linear correlation up to 0.2 IU/mL, we decided to focus on the lower, clinically relevant range of plasma ADAMTS-13 activity by using the above-described assay principle.

### Assay Validation and Comparative Analysis of Patient Samples

In order to determine the lower limit of quantification (LLOQ) of the assay, heat-inactivated pooled normal plasma (PNP; “0” IU/mL calibrator) was analyzed in triplicate during six independent experiments, each also including the five additional plasma ADAMTS-13 calibrators up to 0.20 IU/mL (Figure 2A). MFI values (PE) plus 9 times the SD were defined to reflect the LLOQ as calculated from corresponding interpolation functions. Accordingly, the overall mean (±SD) LLOQ was found to be 0.005 ± 0.003 IU/mL, demonstrating adequate assay sensitivity for assessment of plasma ADAMTS-13 activity with respect to the clinical threshold of 0.1 IU/mL. In addition, assay accuracy and precision were determined by analysis of plasma ADAMTS-13 activity controls (0.15 and 0.05 IU/mL) during three of these experiments. As shown in detail in Table S1, both (mean) intra- and interassay coefficients of variation (CVs) were found to be well below 20%, while relative errors of absolute values did not exceed |6| and |30|% for the higher and lower control target concentrations, respectively. Assay robustness was assessed by varying the time between stopping of the cleavage reactions and flow cytometric analysis (up to 120 min, Figure S5) as well as the analysis of fresh versus once frozen/thawed plasma samples (Figure S6), with only minor impact on assay outcome detected.

**Figure 3:**
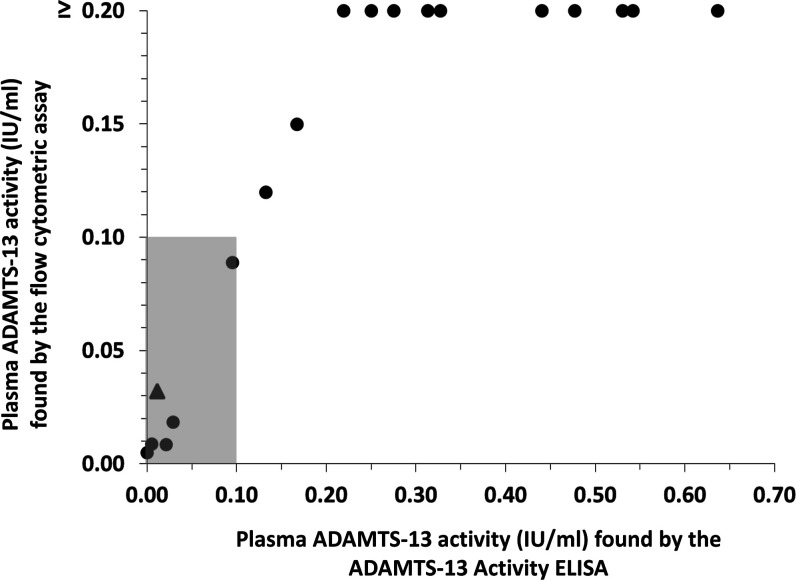
Comparative analysis of patient plasma samples. Samples were analyzed by a commercial ADAMTS-13 activity ELISA used for routine analysis and the flow cytometric assay described in the present paper. The gray box represents the range below the clinical threshold for TTP (<0.1 IU/mL). The triangle represents the sample used for further analysis of assay precision at low plasma ADAMTS-13 activity.

Citrated plasma samples (*n* = 18) from patients with suspected TTP (stored at <−40 °C) were available for comparative analysis by a routinely used commercial ADAMTS-13 activity-ELISA and the flow cytometric assay described here. Results were given as IU/mL of plasma ADAMTS-13 activity and are summarized in Figure 3.

While the quantitative analysis range of the flow cytometric assay was limited due to the reasons discussed above, full agreement of results was found with respect to the clinical threshold for TTP (<0.10 IU/mL). In four of the eight plasma samples that showed an ADAMTS-13 activity below 0.2 IU/mL, antibodies against the enzyme could be detected by ELISA (data not shown). To further assess the precision of the flow cytometric assay in the low-concentration range, one of the patient samples with low plasma ADAMTS-13 activity (0.012 IU/mL as measured by the ADAMTS-13 activity-ELISA, highlighted in Figure 3) was tested in triplicate in three independent experiments. The overall mean (±SD) plasma ADAMTS-13 activity was found to be 0.032 ± 0.0086 IU/mL corresponding to an interassay CV of 27.0%, while the mean intra-assay CV was calculated as 12.8%. These numbers confirm acceptable assay precision in the critical low plasma ADAMTS-13 activity range and further highlight the usefulness of the flow cytometric assay during identification of TTP.

## Conclusions

In order to increase the number of laboratories capable of quantitative plasma ADAMTS-13 activity analysis, we have developed and validated the flow cytometric assay as described here. Flow cytometry is a well-established and widely used technology with a high number of analyzers available in numerous laboratories worldwide.^37,38^ In comparison to complex multiplex cell expression or suspension bead array analysis,^39,40^ the fluorescence detection strategy proposed here is relatively simple. In fact, virtually all flow cytometers currently in use in clinical laboratories could be applicable for quantitative plasma ADAMTS-13 activity testing, with only minor additional laboratory equipment and materials needed. Furthermore, the performance characteristics demonstrated here include a short assay time (80 to 90 min, depending on the number of samples analyzed) and assay robustness, an LLOQ below 0.01 IU/mL, as well as intra- as well as interassay CVs <30% even at low ADAMTS-13 activity levels. In addition, full agreement of results with respect to the clinically relevant 0.1 IU/mL threshold was found when compared to analysis by a well-established, quantitative ELISA. In summary, these preliminary assay characteristics and data provide confidence that the described quantitative, flow cytometric plasma ADAMTS-13 activity assay is deemed to be useful and could in the future be implemented in and successfully validated also by other laboratories.

## Methods

### Key Materials and Flow Cytometer

The human vWF-A2 peptide substrate (vWF73) used in this assay is comprised of amino acids D1596 to R1668, an N-terminal biotin, as well as a C-terminal NH_2_-group for increased nuclease resistance. The molecules were synthesized, purified (>95% purity), lyophilized, and shipped by PSL (Heidelberg, Germany). On site, the material was reconstituted in 10% DMSO in distilled water and stored in aliquots at −80 °C until use. The anti-human vWF-A2 (ADAMTS-13 cleaved)-specific mAb (clone #490628) was ordered from Bio-Techne GmbH (Wiesbaden-Nordenstadt, Germany). In order to prepare this mAb for flow cytometry, the R-PE conjugation kit (Abcam, Berlin, Germany) was used according to the manufacturer’s instructions, and PE-labeled mAbs (PE–mAb) were stored at 4 °C in the dark until use (see the Supporting Methods for details). Dynabeads M-280 streptavidin (Thermo Fisher Scientific, Darmstadt, Germany) were used as a carrier of biotinylated (cleaved) vWF73/PE–mAb complexes for the flow cytometric readout. These beads are superparamagnetic, have a diameter of 2.8 μm, and include streptavidin covalently coupled to the surface. Although easily identifiable by FCS/SSC characteristics, the beads were labeled with Alexa Fluor 647 (“AF647”, see Supporting Methods for details) in order to allow for fluorescence-based gating as previously described.^41^ A Navios EX flow cytometer (Beckman Coulter, Krefeld, Germany) was used for the assay readout. Loaded beads were identified by corresponding FCS/SSC- or AF647-positive events (FL6) while binding of the PE–mAb was assessed by the PE emission pattern (FL2). Further details according to the Minimum Information about Flow Cytometry experiment (MIFlowCyt) standard^42^ can be found in the Supporting Information. The TECHNOZYM ADAMTS13 activity ELISA (“ADAMTS-13 activity-ELISA,” Technoclone, Vienna, Austria) was used for comparative analysis of patient samples as well as for verification of the plasma calibrators and controls described below. The TECHNOZYM ADAMTS13 INH ELISA was used for determination of anti-ADAMTS-13 antibodies. Both assays were performed according to the manufacturer’s instructions using an automated MTP washer (ELx50, Agilent, Waldbronn, Germany) and a multimode MTP reader (Synergy 2, Agilent).

### Assay Optimization and Final Assay Conditions

Optimization of cleavage of biotinylated vWF73 by plasma ADAMTS-13 was based on previous studies on ADAMTS-13 activity with vWF substrates in vitro.^17,18^ This resulted in the following reaction conditions and principles with focus on assay sensitivity: 180 μL of substrate solution (316 nM biotinylated vWF73 in 10 mM Tris-HCl, pH 9.0, 5 mM BaCl_2_, 0.015% Tween 20) was added to 1.5 mL reaction tubes (Eppendorf, Hamburg, Germany) and pre-equilibrated at 37 °C for 10 min using a shaking incubator (Eppendorf). Afterward, 20 μL of plasma samples or calibrators/controls were added, and the mixtures were incubated at 37 °C under shaking (1100 rpm) for 30 min (see the Supporting Methods for details on cleavage reactions performed in the presence of protease inhibitors). In order to stop the cleavage reactions, 800 μL of stopping solution (DPBS, pH 7.4, 25 mM EDTA) was added, and tubes were vortexed and stored at room temperature (RT) until the final assay readout. For the flow cytometric analysis of ADAMTS-13-mediated cleavage of biotinylated vWF73, 95 μL of measuring buffer (DPBS, pH 7.4, 5 mM EDTA) was pipetted to brown (light shielded) 1.5 mL reaction tubes (Eppendorf), and 5 μL of the cleavage reaction mixture was added. After mixing, 10 μL of AF647-labeled beads (1000/μL in DPBS, pH 7.4, 0.1% BSA > yielding 10,000 beads/reaction) and 2 μL of PE–mAb (∼330 nM in DPBS, pH 7.4, 0.1% BSA, 2 mM NaN_3_ > 0.66 pmol/reaction, Table S2) were added, and the mixtures were incubated for assembly at RT while shaking (1100 rpm) for 20 min. Subsequently, further 400 μL of measuring buffer was added to each tube, and the mixtures were transferred to 12 × 75 mm polypropylene tubes (Beckman Coulter) for flow cytometric analysis. For each reaction, 1000 AF647-labeled bead events were recorded (FL6) and associated binding of PE–mAb was assessed by PE mean fluorescence intensity (FL2).

### Preparation of Calibrators and Controls

The WHO international standard for plasma ADAMTS-13 antigen and activity (WHO 1st International Standard ADAMTS13 Plasma, NIBSC code: 12/252)^43^ and PNP were used for preparation of plasma ADAMTS-13 calibrators and controls. In brief, PNP was prepared from whole blood [anticoagulated with sodium citrate (10.5 nM final concentration)] obtained from four healthy blood donors, who gave informed written consent, and frozen in aliquots of 1 mL at −40 °C until used. Parts of the PNP were heat-inactivated at 56 °C for 40 min in order to eliminate all ADAMTS-13 activity. Analysis of PNP as well as heat-inactivated PNP by the ADAMTS-13 activity-ELISA revealed plasma ADAMTS-13 activities of 1.03 and <0.005 IU/mL (lower limit of detection), respectively. The heat-inactivated PNP served as the plasma matrix diluent for the PNP to prepare relevant controls (0.15 and 0.05 IU/mL) around the clinical threshold of 0.1 IU/mL. The heat-inactivated PNP was also used for dilution of the WHO plasma ADAMTS-13 standard to prepare (initial) calibrators: *n* = 9; 0.91 IU/mL (nominal activity of the WHO plasma ADAMTS-13 standard after reconstitution) down to “0” IU/mL (HI PNP). All calibrators and controls were aliquoted and stored at −80 °C until used.

## Supporting Information Available

Labeling and characterization of the anti-human vWF-A2 (ADAMTS-13 cleaved)-specific mAb; labeling of Dynabeads M-280 streptavidin with Alexa Fluor 647; evaluation of assay specificity; and MIFlowCyt summary (PDF)
