# Supplementary material for: Assay for ADAMTS-13 Activity with Flow Cytometric Readout
Source: ACS Omega. 2022 Aug 24;7(35):30801–6. doi: 10.1021/acsomega.2c02077 (PMC9453954; doi:10.1021/acsomega.2c02077)

An Assay for ADAMTS-13 Activity with Flow Cytometric Readout

Jens Müller*, Nasim Shahidi Hamedani, Hannah L. McRae, Heiko Rühl, Johannes Oldenburg, Bernd Pötzsch

Institute of Experimental Hematology and Transfusion Medicine, University Hospital Bonn, Bonn, Germany

Supporting Information

Supporting Methods (Page S2-S3)

Supporting Table and Figures (Page S4-S9)

Supporting MIFlowCyt Summary (Page S10-S14)

Supporting Methods

Labeling and characterization of the anti-human vWF-A2 (ADAMTS-13 cleaved)-specific mAb (clone #490628)

In order to apply the anti-human vWF-A2 (ADAMTS-13 cleaved)-specific mAb for flow cytometry, the R-Phycoerythrin (PE) Conjugation Kit (cat. no. ab102918) Abcam, Berlin, Germany) was used according to the manufacturer's instructions. Since there is only a minor change of input volume of the sample and also no purification step needed when using this kit, the concentration of the labeled antibody was set according to the original input concentration (500 µg/ml = 3,33 µM). For preparation of a working solution, the labeled antibody was further diluted 1 in 10 using DPBS pH 7.4, 0.1% BSA, 2 mM NaN_3 and stored at 4°C until use.

Assay optimization included testing of different quantities of the PE-labeled antibody per reaction. With respect to best possible assay sensitivity, corresponding results indicated an optimal signal-to-noise ratio (S/N, 1% / 0% plasma ADAMTS-13 activity) when using 2 µl of the PE-labeled mAB (0.66 pmol/reaction, Table S2). This amount of PE-mAb was therefore used during all experiments described in the paper.

In order to assess the degree of labeling when using the (PE) Conjugation Kit, another batch of the anti-human vWF-A2 (ADAMTS-13 cleaved)-specific mAb was labeled along with an anti-protein C antibody and non-reducing sodium-dodecyl-sulfate polyacrylamide-gel-electrophoresis (SDS-PAGE) followed by fluorescence readout were performed. Silver staining was used for further characterization. In brief, samples containing one µg (6.67 pmol) of either non-conjugated mAb or PE-mAb were mixed with the same volume of 2x Laemmli buffer and run on 4–15% Mini-PROTEAN TGX Stain-Free Gels (BioRad, Munich, Germany). Detection of PE fluorescence and visualization of silver staining (SilverXpress sliver staining kit, ThermoFisher Scientific, Darmstadt, Germany) was done using a GelDoc Imaging System (Bio-Rad, Munich, Germany).

As shown in Figure S1, a significant proportion of the anti-human vWF-A2 (ADAMTS-13 cleaved)-specific mAb introduced remained unlabeled. Most of the PE-conjugated mAbs obviously exhibited one PE-molecule,Evaluation of the novel PE-mAb preparation revealed good performance in the low plasma ADAMTS-13 activity range, indicating reproducible and sufficient performance of the applied PE-labeling kit with respect to the assay described here. However, the assay may be further improved by using a PE-mAb preparation with higher degree of labelling.

Labeling of Dynabeads M-280 Streptavidin with Alexa Fluor 647

One hundred twenty-five µl (1.25 mg) Dynabeads M-280 Streptavidin (approx. $6.5*10^7$ beads/mg) were pipetted into a 1.5 ml reaction tube and washed two times with 1 ml of 0.2 M sodium carbonate buffer ('carbonate buffer', pH 8.3). Subsequently, beads were mixed with

800 µl carbonate buffer and 200 µl aliquots of the solution (primed beads) added to novel 1.5 ml reaction tubes. Alexa Fluor 647 NHS ester (MW: 1250, #A37573 [ThermoFisher, Darmstadt, Germany], Lot 2123596: 99% purity > 99 µg / vial) was dissolved in 50 µl DMSO, yielding a 1.58 mM solution ($9.5*10^{14}$ molecules/µl) that was further diluted in carbonate buffer and added to the primed beads to achieve $5.5*10^8$, $5.5*10^7$, $5.5*10^6$ and 0 (control) molecules/bead in a final volume of 400 µl each. After shaking for 30 min at room temperature (RT) in the dark, 50 µl of 1 M Tris-HCl (pH 7.6) were added to each tube and reactions incubated for a further 10 min at RT on a shaker. Subsequently, AF647-labelled beads were washed two times with 1 ml DPBS/BSA (pH 7.4, 1 mg/ml BSA), taken up in 200 µl of DPBS/BSA containing 5 mM NaN_3 (yielding ~ 100.000 labelled beads / µl), and stored at 4°C in the dark until used. Assessment of fluorescence pattern (Navios EX, AF647(FL6)/SSC) revealed distinct MFI values for each AF647-labeled bead fraction. The AF647-labled beads prepared by incubation with $5.5*10^6$ Alexa Fluor™ 647 NHS ester molecules / bead were ultimately used for all further analysis described in the manuscript.

Evaluation of assay specificity

Pierce Protease Inhibitor Mini Tablets, EDTA-free (ThermoFisher Scientific, Cat. No. A32955) were used to evaluate the specificity of the assay. The tablets contain broad-spectrum protease inhibitors (AEBSF, aprotinin, bestatin, E-64, leupeptin, and pepstatin A) and were dissolved in the used substrate buffer (10 mM Tris-HCl, pH 9.0, 5 mM $BaCl_2$, 0.015% Tween 20) according to the manufacturers recommendations. Due to the EDTA-free formulation, the protease inhibitor mix is not effective against metalloproteases. Analysis of plasma calibrators and patient samples (< 0.2 IU/ml ADAMTS-13 activity) was done in parallel, in the absence and presence of the protease inhibitor mix. Obtained results are shown in Figure S4.

Supporting Table and Figures

Table S1. Determined assay reproducibility and precision.

Plasma controls	Run #1		Run #2		Run #3		Coefficients of variation (CV)	
**ADAMTS-13 (IU/ml)**	**mean (IU/ml)**	**RE (%)**	**mean (IU/ml)**	**RE (%)**	**mean (IU/ml)**	**RE (%)**	**intra-assay CV (%)[a]**	**inter-assay CV (%)**
**0.150**	0.150	0.0	0.151	0.7	0.141	-6.0	3.79	3.59
**0.050**	0.046	-8.0	0.035	-30.0	0.052	4.0	4.75	16.34

[a] mean value, RE: relative error

Table S2. Assay optimization: different quantities of PE-labeled antibodies per reaction.

	PE-labeled mAb (MFI)			PE-labeled mAb (S/N)		
**Plasma ADAMTS-13 (IU/ml)**	**0.33 pmol / reaction**	**0.66 pmol / reaction**	**1.0 pmol / reaction**	**0.33 pmol / reaction**	**0.66 pmol / reaction**	**1.0 pmol / reaction**
**0.2**	13.10	15.40	29.10	17.04	18.12	23.28
**0.15**	10.20	12.10	19.56	13.26	14.24	15.65
**0.1**	5.89	9.55	16.50	7.66	11.24	13.20
**0.05**	3.12	5.10	7.56	4.06	6.00	6.05
**0.01**	1.12	1.43	1.67	1.46	1.68	1.34
**0**	0.77	0.85	1.25	1.00	1.00	1.00

MFI: mean fluorescence intensity; S/N: signal to noise ratio

Figure S1

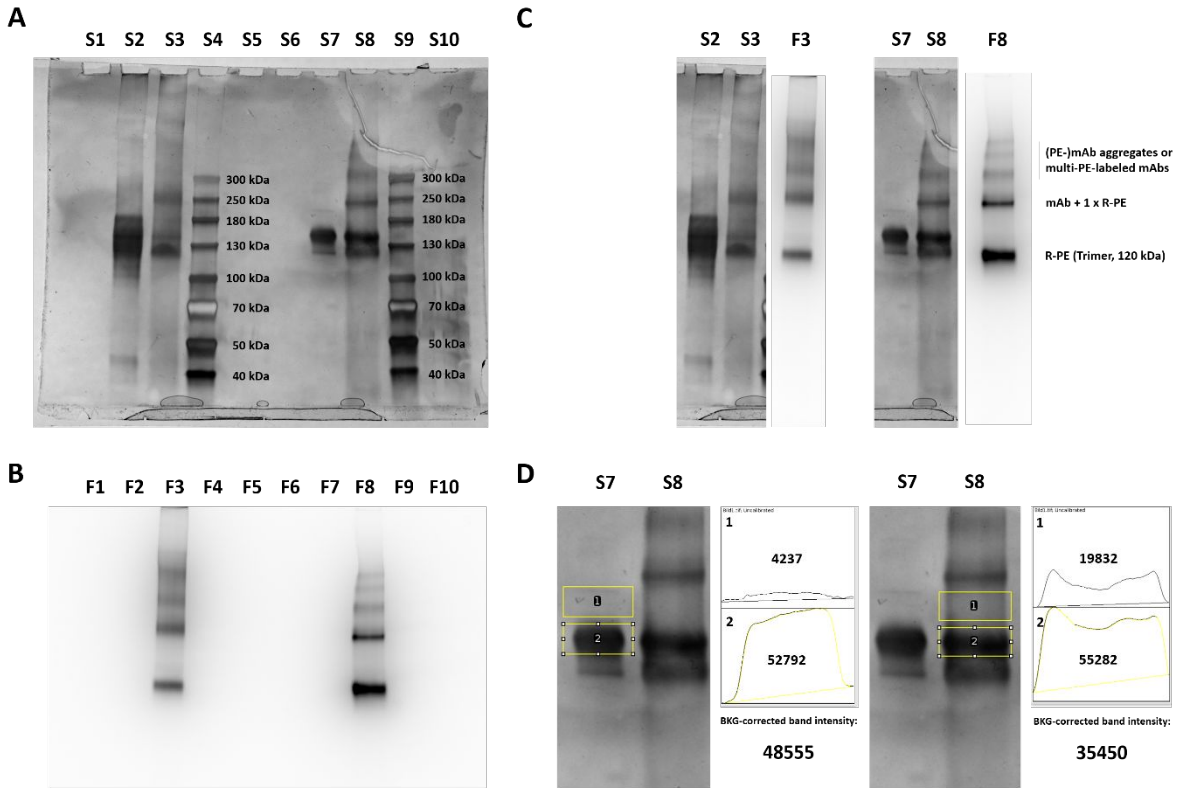

E

Plasma ADAMTS-13 (IU/ml)	PE MFI (0.66 pmol/reaction)
**0.25**	20.90
**0.125**	11.00
**0.06**	5.70
**0.03**	2.70
**0.015**	1.50
**0.0078**	1.00
**0**	0.83

Characterization of the PE-labeled anti-human vWF-A2 (ADAMTS-13 cleaved)-specific mAb. A) Silver staining. Lane S7: unlabeled mAb, Lane S8: PE-labeled mAb, Lane S9: Protein marker (Spectra™ High Range Protein Ladder, cat. No. #26625, ThermoFisher Scientific). The same order of loading was applied for characterization of an anti-protein C antibody labeled in parallel (lane S2 to S4). B) PE-fluorescence emission pattern of the same gel. C) Alignment of bands of silver staining und PE-fluorescence indicate the application of (αβ)3 trimers (120 kDa) as functional units of R-PE in the labeling reactions (Jiang et al. Proteins. 1999; 34: 224-31). D) Background-corrected band intensities of unlabeled mAb before (S7) and after (S8) PE-labelling indicate that approx. 75% of the mAb-molecules remained unlabeled. Analysis was done using the browser version of ImageJ run in Google Chrome (https://ij.imjoy.io). E) PE MFI values obtained from analysis of a serial dilution of the WHO international standard for plasma ADAMTS-13 antigen and activity (NIBSC code: 12/252) in heat-inactivated pooled normal plasma. The PE-labeled mAb shown in lane S8/F8 was used for analysis.

Figure S2

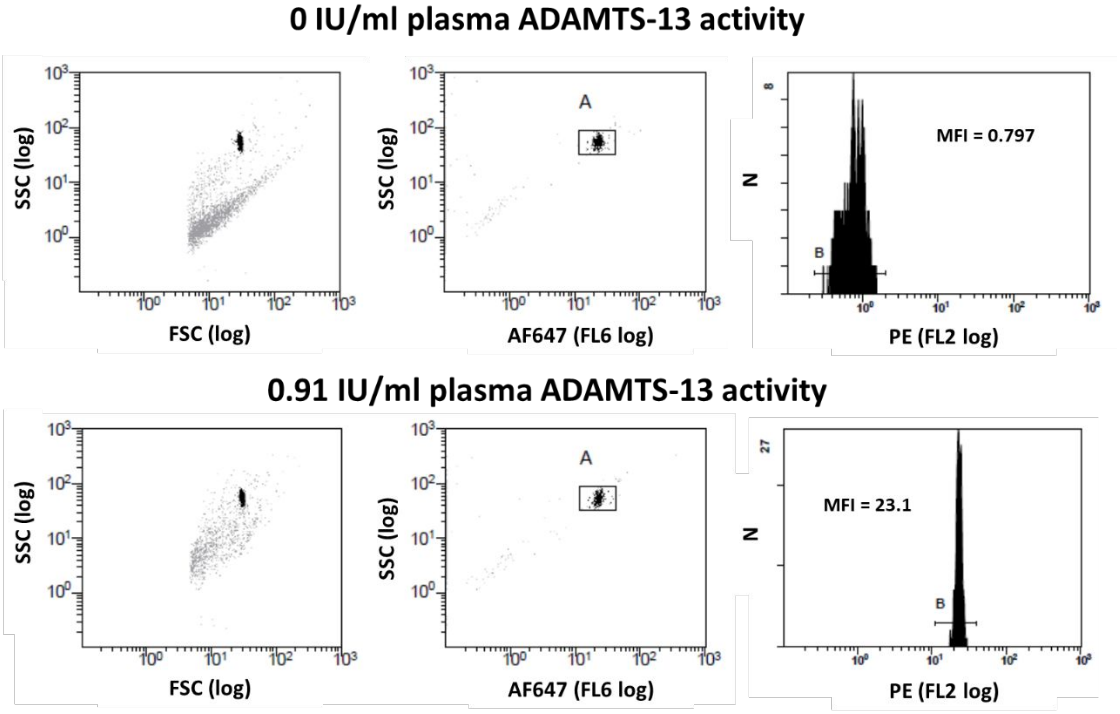

Figure S2. No agglutination of AF647-labeled Dynabeads M-280 Streptavidin even at high plasma ADAMTS-13 activity. Both the FSC/SSC- and FL6/SSC patterns demonstrated no agglutination of loaded and analyzed beads, even at high plasma ADAMTS-13 input concentrations. Plots were taken from analysis shown in Figure 2. Upper row: no plasma ADAMTS-13 activity, lower row: 0.91 IU/ml plasma ADAMTS-13 activity as contained in the used WHO standard preparation.

Figure S3

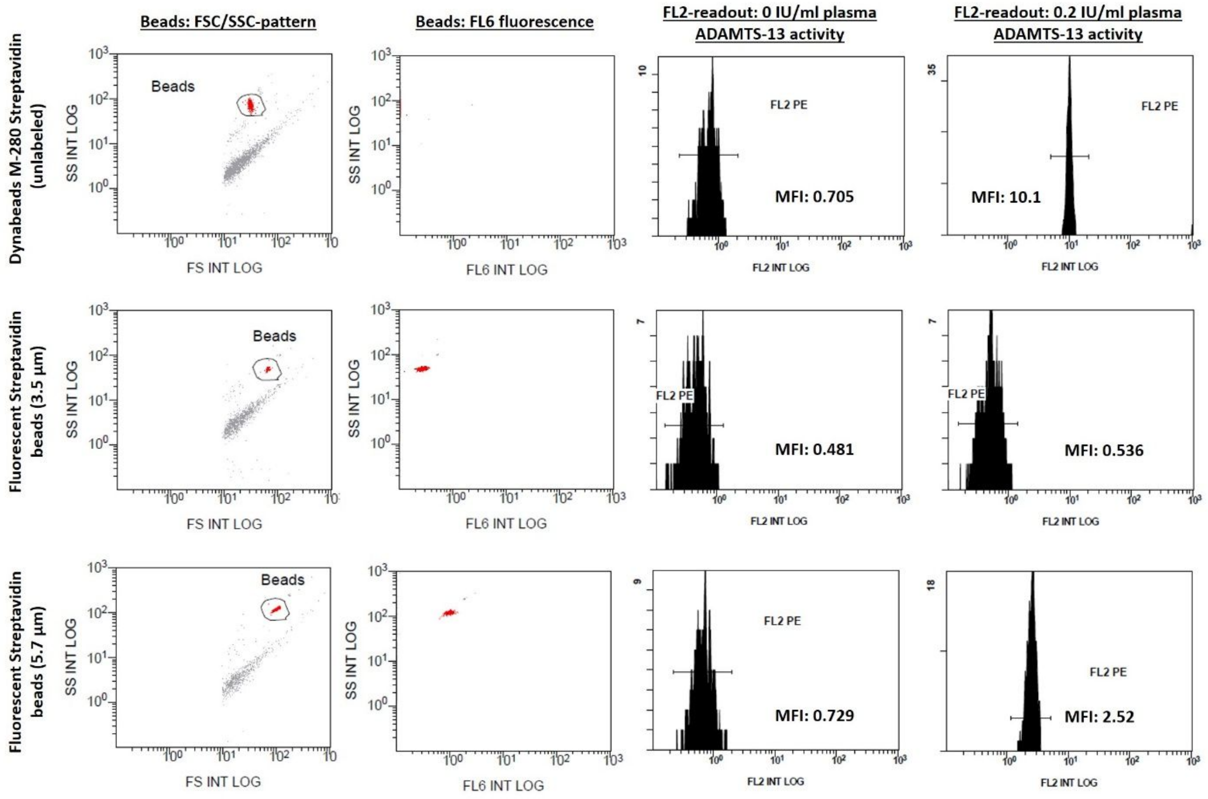

Figure S3. Assessment of Dynabeads M-280 Streptavidin (2.8 µm diameter, upper row) vs. commercially available, fluoresecent streptavidin-coated beads with diameters of 3.5 µm (middle row) and 5.7 µm (lower row). Please note that these experiments were performed at an early stage under use of different cytometer settings, leading to lower absolute MFI values, especially at higher plasma ADAMTS-13 activities than found later and reported in the main text.

Figure S4

A

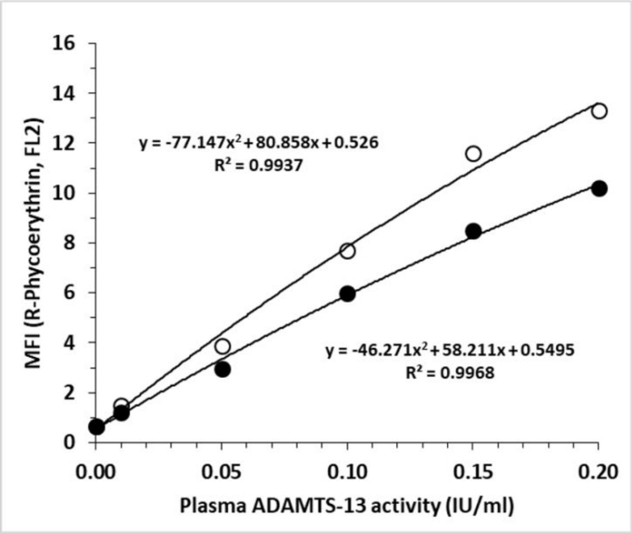

B

	MFI (R-Phycoerytrhin, FL2)	
Sample #	w/o inhibitor mix	w inhibitor mix
1	12.00	8.29
2	2.47	1.90
3	0.65	0.65
4	6.74	5.40
5	3.18	2.05
6	1.56	1.32

C

	Plasma ADAMTS-13 activity (IU/ml)	
Sample #	w/o inhibitor mix	w inhibitor mix
1	0.169	0.151
2	0.025	0.024
3	0.002	0.001
4	0.084	0.090
5	0.034	0.026
6	0.013	0.013

Figure S4. Assessment of assay specificity. Plasma calibrators (A) and patient samples (B, C) were analyzed in parallel, in the absence (A, open symbols) and presence (A, closed symbols) of broad-spectrum protease inhibitors (see Supporting Methods for details). While absolute MFI-values were generally found to be lower in the presence of the protease inhibitor mix (A, B), calculated quantitative ADAMTS-13 activity values were comparable (C). These findings indicate generic rather than specific effects of the protease inhibitors on substrate (biotinylated vWF73) cleavage.

Figure S5

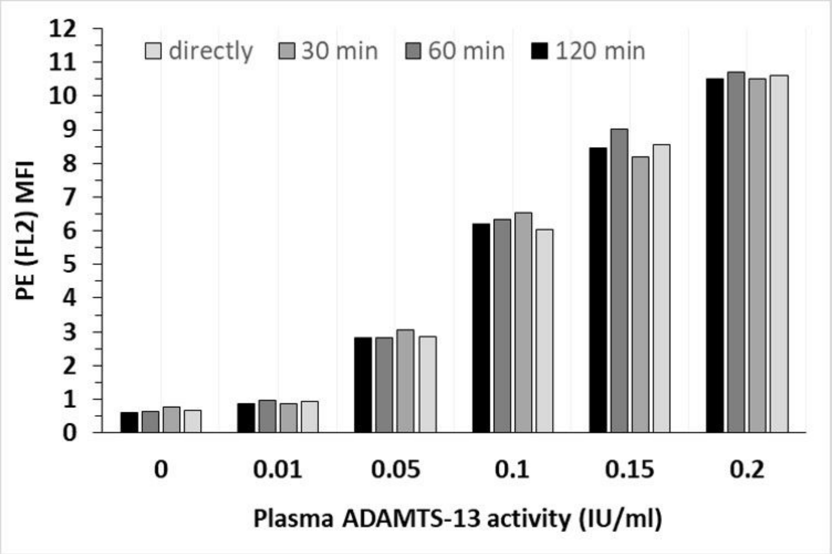

Figure S5. Assay robustness. Assessment of different time intervals (as indicated) between stopping of the cleavage reactions by EDTA and flow cytometric assay readout. Please note that these experiments were performed at an early stage under use of different cytometer settings, leading to lower absolute MFI values especially at higher plasma ADAMTS-13 activities than found later and reported in the main text.

Figure S6

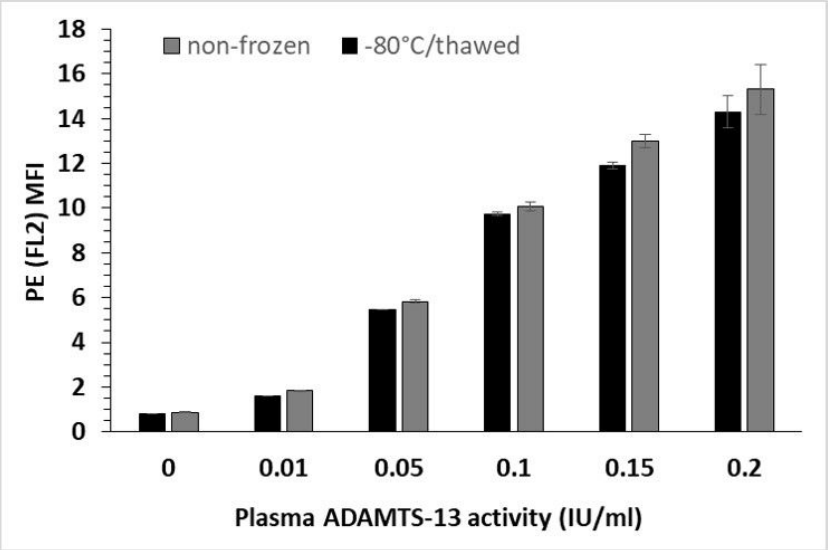

Figure S6. Assay robustness. Assessment of the effect of freezing/thawing of plasma samples on assay results. The WHO plasma ADAMTS-13 standard was reconstituted and diluted in heat-inactivated PNP to yield the indicated activities (calibrators). Subsequently, samples were analyzed in parallel to previously prepared and frozen (-80°C) / thawed (37°C) calibrators.

Minimum Information about the Flow Cytometry Experiment (MIFlowCyt)

1. Experiment Overview

1.1 Purpose

The purpose of the study is to establish a flow cytometric method that allows the determination of ADAMTS-13 activity in citrated plasma samples.

1.2 Keywords

Thrombotic microangiopathy (TMA), thrombotic thrombocytopenic purpura (TTP), citrated plasma, ADAMTS-13, flow cytometry.

1.3 Experiment Variables

An N-terminally biotinylated minimal peptide motive for ADAMTS-13 (vWF73) was used as substrate for determination of ADAMTS-13 activity in citrated plasma samples. Cleavage rates of the substrate under optimized assay conditions were determined by incubation of (cleaved) vWF73 with Alexa Fluor 647-labeled Dynabeads M-280 Streptavidin and a PE-labeled monoclonal antibody (mAb) that specifically recognizes the ADAMTS-13 cleavage site. After incubation, the reaction mixture was analyzed by flow cytometric readout, with Alexa Fluor 647-labeled beads gated by specific fluorescence pattern (FL6) and the amount of bound mAB determined by PE (FL2) mean fluorescence intensities (MFI). Assay calibrators were prepared by dilution of the WHO international standard for plasma ADAMTS-13 antigen and activity (WHO 1st International Standard ADAMTS13 Plasma, NIBSC code: 12/252) into heat-inactivated citrated pooled normal plasma (PNP, in-house preparation). Plasma calibrators were prepared by dilution of characterized PNP into heat-inactivated PNP. Validation of assay characteristics included determination of the lower limit of quantification (LLOQ) as well as assay reproducibility and precision. Plasma samples (citrate [10.5 mM]) of patients with suspected TTP were assayed to verify the clinical applicability of the proposed method.

1.4 Organization

1.4.1. Name

University of Bonn Medical Center, Institute of Exp. Haematology and Transfusion Medicine

1.4.2. Address

Venusberg-Campus 1, 53127 Bonn, Germany

1.5 Primary Contact

1.5.1. Name: Jens Müller

1.5.2. Email: jens.mueller@ukbonn.de

1.6 Date

Experiments were performed in 2018-2021.

1.7 Conclusions

The assay performance characteristics evaluated demonstrated a sufficient LLOQ as well as acceptable assay reproducibility and precision. Compared to the results obtained with a commercially available

quantitative ADAMTS-13 activity ELISA, analysis of 18 citrated plasma samples from patients with suspected TTP revealed full agreement of results with respect to clinical decision limits. Thus, the assay appears to be applicable for routine clinical analysis.

1.8 Quality Control Measures
The flow cytometer optical alignment and fluidics system were calibrated / checked using Flow-Check Pro Fluorospheres (Beckman Coulter) according to the manufacturers´ instructions. Plasma ADAMTS-13 controls above and below the clinical decision limit (0.1 IU/ml plasma ADAMTS-13 activity) were included in each run.

2. Flow Sample / Specimen Details

2.1 Sample/Specimen Material Description

2.1.1 Biological Samples

WHO international standard for plasma ADAMTS-13 antigen and activity (WHO 1st International Standard ADAMTS13 Plasma, NIBSC code: 12/252).

Citrated pooled normal plasma (PNP) was prepared from citrate-anticoagulated whole blood obtained from 4 healthy blood donors who gave informed written consent. PNP was frozen in aliquots of 1 ml at -40°C until use. A portion of the PNP were heat inactivated to eliminate all ADAMTS-13 activity in order to allow for preparation of plasma ADAMTS-13 calibrators and controls.

Whole blood from 18 patients with suspected TTP was drawn from an antecubital vein into citrate-tubes (Sarstedt, Nümbrecht, Germany; final sodium citrate concentration: 10.5 mM) and transferred to our laboratory for routine plasma ADAMTS-13 activity analysis. Aliquots of citrated plasma prepared by centrifugation were stored at ≤ -40°C to be used for additional investigation by the flow cytometric assay described here.

2.1.2 Evironmental Samples
N/A

2.1.3 Other Samples
N/A

2.2 Sample Characterisitics

The range of ADAMTS-13 activity in the 18 patient plasma samples (as assessed by routine ELISA) was found to be < LLOQ to 0.636 IU/ml. The WHO 1st International Standard ADAMTS13 Plasma contained 0.91 IU/ml plasma ADAMTS-13 activity. The prepared PNP showed an ADAMTS-13 activity of 1.03 IU/ml. Prepared calibrators covered plasma ADAMTS-13 activities from 0.91 down to 0 IU/ml. Prepared controls flanked the clinical decision limit of 0.1 IU/ml (0.15 IU/ml and 0.05 IU/ml).

2.3 Sample Treatment Description

180 µl of substrate solution (316 nM biotinylated vWF73 in 10 mM Tris-HCl, pH 9.0, 5 mM BaCl2, 0.015% Tween 20) were added to 1.5 ml reaction tubes (Eppendorf, Hamburg, Germany) and pre-equilibrated at 37°C for 10 min using a shaking incubator (Eppendorf). Afterwards, 20 µl of plasma samples or calibrators / controls were added and mixtures incubated at 37°C under shaking (1100 rpm) for 30 min. In order to stop the cleavage reactions, 800 µl of stopping solution (DPBS, pH 7.4, 25 mM EDTA) were added, tubes vortexed and stored at room temperature. For subsequent flow cytometric analysis, 95 µl of measuring buffer (DPBS, pH 7.4, 5 mM EDTA) were pipetted to brown (light shielded) 1.5 ml reaction tubes (Eppendorf) and 5 µl of the cleavage reaction mixture added. After mixing, 10 µl of AF647-labeled beads (1,000/µl in DPBS, pH 7.4, 0.1% BSA > yielding 10,000 beads/reaction), and 2 µl of PE-mAb (~ 330 nM in DPBS, pH 7.4, 0.1% BSA, 2 mM NaN3 > 0.66 pmol/reaction) were added and mixtures incubated for assembly at RT under shaking (1,100 rpm) for 20 min. Subsequently, a further 400 µl of measuring buffer were added to each tube and the mixtures transferred to 12 x 75 mm polypropylene tubes (Beckman Coulter) for flow cytometric analysis. For each reaction, 1,000 AF647-labeled bead events were recorded (FL6) and associated binding of PE-mAb assessed by PE MFI (FL2).

2.4 Fluorescence Reagent Description

Each staining reaction / all tubes were analyzed according to the following scheme:

**(Optical) Detector**	**FSC**	**SSC**	**FL2**	**FL6**
**Reporter Dye**	-	-	PE	Alexa Fluor 647
**Scale**	log	log	log	Log
**Threshold value**	5	-	-	-

Compensation was performed as follows: **N/A** (no compensation necessary).

The following reagents have been used:

**Reagent**	**Label**	**Manufacturer**	**Clone**	**Cat#**
B-vWF73*	N-terminal Biotin	PSL Heidelberg	N/A	N/A
anti-human vWF (cleaved)	PE**	Bio-Techne GmbH	490628	MAB27642
** PE Conjugation Kit	PE	Abcam	N/A	ab102918
Dynabeads M-280 Strept.	AF647***	Thermo Fisher	N/A	11205D
***Alexa Fluor 647 NHS-Ester	AF647	Thermo Fisher	N/A	A20006

* ADAMTS-13 minimal substrate motive

3. Instrument Details

3.1 Instrument Manufacturer
Beckman Coulter

3.2 Instrument Model
Navios EX; S/N: BA14209 / BA14209

3.3 Instrument Configurations and Settings

3.3.1 Flow Cell and Fluidics
Original configuration, instrument has not been altered.

3.3.2 Light Sources
Original configuration:
- Blue Solid-state diode laser, 488 nm, 22 mW
- Red Solid-state diode laser, 638 nm, 25 mW

3.3.3 Excitation Optics Configurations
Original configuration, instrument has not been altered.

3.3.4 Optical Filters
Original configuration, all filters came with instrument (May, 2018).

3.3.5 Optical Detectors
Original configuration, instrument has not been altered.
For the present study, detector voltages / gains have been set to:
FSC: 250 V, Gain = 1.0, Threshold: 5
SSC: 250 V, Gain = 1.0
FL2: 325 V, Gain = 1.0
FL6: 436 V, Gain = 1.0

3.3.6 Optical Paths
Original configuration, instrument has not been altered.

4. Data Analysis Details

4.1 List-mode Data Files
Original data files can be obtained from the corresponding author of this work.

4.2 Compensation Description
N/A

4.3 Data Transformation Details

4.3.1 Purpose of Data Transformation
Visualization of data and gating.

4.3.2 Data Transformation Description
The original software, that came with the instrument, was used ("Cytometry List Mode Data Acquisition & Analysis Software" [Navios EX Cytometer 2.0)). For analysis, data were displayed as follows (x-axis/y-axis; also cf. point 4.4.3 below): [FSC (log) / SSC (log)] | FL6 (log) /SSC (log) > FL2 (log) / #events (linear).

4.4 Gating (Data Filtering) Details

After assay optimization, the same gating strategy was used for all samples / data files. See point 4.4.1 for details.

4.4.1 Gate Description

Bead-events (Alexa Fluor 647 labeled Dynabeads M-280 Streptavidin) were identified / gated by FL6/SSC dot plot characteristics (cf. Panel A in 4.4.3 below). Gated events were further assessed in a FL2 histogram plot with respect to PE MFI (cf. Panel B in 4.4.3 below).

4.4.2 Gate Statistics

A typical run including the analysis 0f 1,000 bead events (FL6) per sample. PE (FL2) MFI values were found to be between approx. 0.9 (0 IU/ml plasma ADAMTS-13 activity) and 15-16 (0.2 IU/ml).

4.4.3 Gate Boundaries

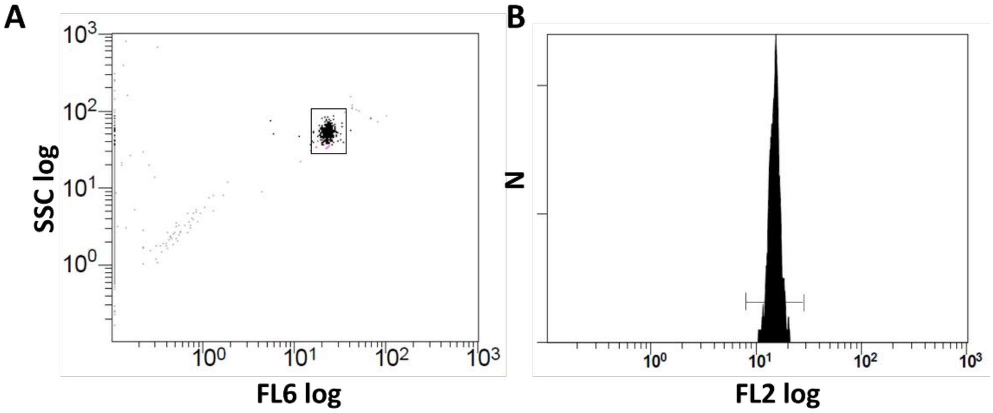

Supplement: Supplementary file 1 — ao2c02077_si_001.pdf [file ao2c02077_si_001.pdf]
